# Anterior spinal separation surgery to allow for stereotactic body radiotherapy: a novel approach permitting radical oncological treatment of oligometastatic disease

**DOI:** 10.1093/jscr/rjad244

**Published:** 2023-05-13

**Authors:** Priyanshu Saha, Bisola Ajayi, Pawan Minhas, Darren F Lui

**Affiliations:** Department of Medicine, St. George’s University of London, London, UK; Department of Complex Neurosurgery, Atkinson Morley Wing, St. George’s NHS Foundation Trust, London, UK; Department of Complex Neurosurgery, Atkinson Morley Wing, St. George’s NHS Foundation Trust, London, UK; Department of Complex Neurosurgery, Atkinson Morley Wing, St. George’s NHS Foundation Trust, London, UK

## Abstract

The treatment of spinal cancers has rapidly evolved in the past decade. Often the treatment for spinal metastases required highly morbid surgeries and with palliative outcomes. However, a paradigm shift in surgical oncology has allowed spinal metastases treatment to have curative results. In the state of oligometastatic disease (OMD), the accompaniment of Stereotactic Body Radiotherapy (SBRT) as a primary modality or adjuvant treatment to surgery has been shown to excellent survival outcomes, lower morbidities and better pain management. This case report illustrates a novel approach to the treatment of spinal OMD utilizing anterior spinal separation surgery with a custom carbon fibre vertebral body replacement cage followed by postoperative SBRT with excellent radio-oncological outcomes over 30-month follow-up.

## INTRODUCTION

Oligometastatic disease (OMD) has been described as a distinct intermediary stage between localized and systemic metastatic disease [1]. Although there aren’t specific biomarkers to differentiate OMD and polymetastatic disease, imaging is utilized as diagnostic purposes and further classification is done based off tumour characteristics and patient history of OMD. Metachronous OMD is described as oligometastatic recurrence that occurs at least 3 months after initial primary OMD. It is furthermore to have metastasized to one or several organs with less than five lesions in a well-controlled primary diseased state [2]. However current NHS guidelines exclude Stereotactic Body Radiotherapy (SBRT) if there are more than three metastases. Historically spinal metastases have been treated palliatively. En bloc surgery for oligometastatic spinal disease is possible but does have significant morbidity [3]. Conventionally, separation surgery includes posterior spinal stabilization with limited tumour resection with 2 mm clearance to the thecal sac to allow safe margins of SBRT [4]. Posterior separation surgery has been shown to be effective in reducing local recurrence [5]. Posterior instrumentation can be technically difficult particularly at the cervical-thoracic junction. We describe the first case of anterior separation surgery allowing tumour clearance of >2 mm from the spinal cord with a custom carbon fibre vertebral body replacement (VBR) cage permitting optimal SBRT planning and delivery for radical oncological surgery. This manuscript is written following the CARE checklist and has written consent from the patient to utlise her operative imaging for research.

## CASE REPORT

A 46-year-old Caucasian female with history of breast cancer and mastectomy attended her annual blood test that revealed elevated Liver Function Test (LFT) and tumour marker hence a referral was made. A solitary metachronous breast T2 oligometastatic lesion was discovered on computed tomography (CT) scan. The patient did not present with any other comorbidities. Spinal Instability Neoplastic Score (SIN) was determined, the location was junctional (C7-T2), pain free, a lytic bone lesion was found on CT with normal alignment and no vertebral collapse. The patient also did not present with any neurological deficit. She was American Society of Anesthesiologists (ASA) grade 3. She had a Tokuhashi score of 14 hence indicating she would live more than a year [6]. The revised Tokuhashi with a Tomita score of 2 indicated for excision surgery [7, 8]. This type of surgery has high morbidity involved. To avoid morbidity, separation surgery with postoperative SBRT was opted. A Bilsky Epidural Spinal Cord Compression (ESCC) score of 2 and the Neurologic Oncologic Mechanical and Systemic framework indicated the patient was suitable for separation surgery. [4] Although the SIN score was 6 indicating that the patient should not be operated on and most likely be treated palliatively, [9, 10] separation surgery was decided for the patient due to the patient’s young age and systemic suitability. Primary stereotactic spinal radiosurgery was not an option due to the ESCC score and the risk of radiation myelitis. This case report illustrates a novel approach to the treatment of spinal OMD utilizing anterior separation surgery with a custom carbon fibre VBR cage followed by postoperative SBRT. Due to several contraindications of posterior separation surgery and the morbidity associated with radical surgery; anterior separation surgery was opted for utilizing the philosophy of Enneking radical surgery despite Enneking inappropriate resection.

**Figure 1:**
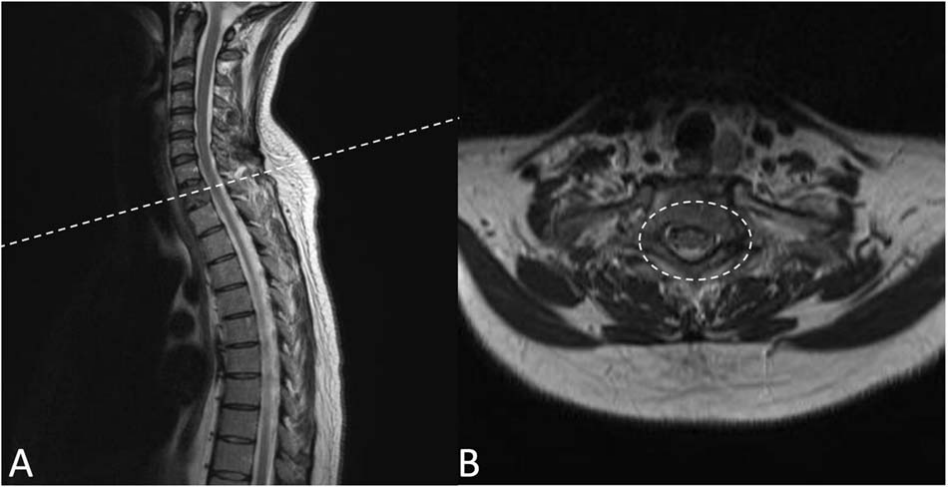
Preoperative MRI Scan December 2019. (**A**) Sagittal view of oligometastatic lesion at T2 vertebral body. (**B**) ESCC grade 2 determined as there is spinal cord compression, but with cerebrospinal fluid visible around the cord.

**Figure 2:**
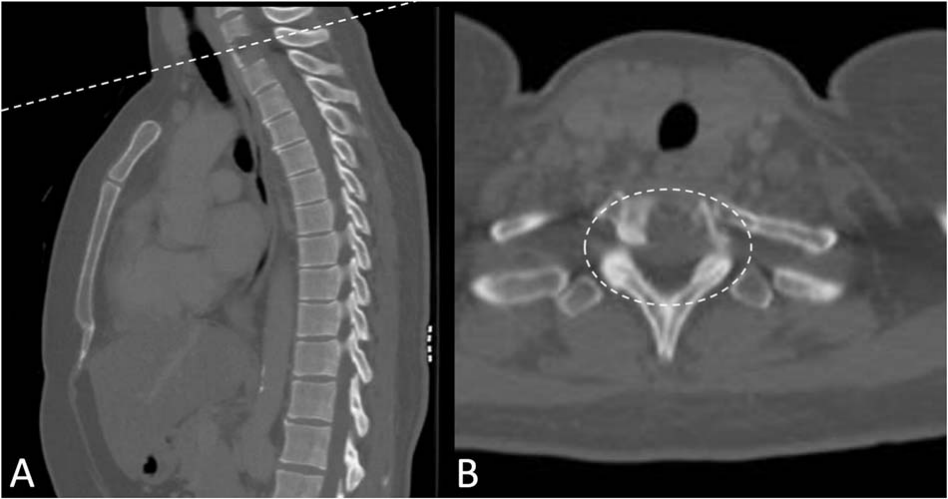
Preoperative CT Scan December 2019. Sagittal view of oligometastatic lesion at T2 vertebral body. (**A**) Sagittal view of oligometastatic lesion at T2 vertebral body. (**B**) Transverse view determined ESCC grade 2 as there is spinal cord compression, but with cerebrospinal fluid visible around the cord.

Preoperative magnetic resonance imaging (MRI) and preoperative CT scans helped determine Tokuhashi, Tomita and ESCC scores, which was vital to plan treatment for this patient (Figs 1 and 2). The occurrence of the lesion occurring in T2 indicated an anterior approach. This would be safer than a posterior approach as the ESCC grade of 2 made it surgically challenging to resect tumour and lay instrumentation around the spinal cord. An anterior approach also avoids cervico–thoracic junction fusion.

Anterior T1 Corpectomy Separation Surgery with Carbon Fibre Cage and Plate Utilizing the CT, a custom-made carbon fibre VBR was manufactured bespoke to the patient’s anatomy. An Anterior Smith Robinson primary approach was undertaken to perform the corpectomy. [11] Intraoperative X-ray was done to confirm the level. A careful piecemeal corpectomy was performed at T1 and stabilized using a custom carbonfibre VBR and a custom Carbon fibre plate. Screw fixation allowed Anterior instrumentation from C7 to T2. The surgical procedure was commenced with a blunt dissection made long the carotid sheath and opened along the carotid artery. This led to excellent exposure of C7-T2. A discectomy was performed at C7/T1 and T1/T2 and the anterior wall of the T1 vertebral body was removed. Diathermy tumour resection was done to ablate and remove the tumour from the inner aspects of the body and the posterior longitudinal ligament. Enneking principles of resection were respected as much as possible. Large sections of the tumour were resected piecemeal and canal soft tissue tumour was excised macroscopically. The custom carbonfibre VBR was inserted under and stabilized with four 14 mm Titanium screws converging to the centre and divergent away from the plate superiorly and inferiorly for rigidity. Satisfactory image intensifier X-rays.

**Figure 3:**
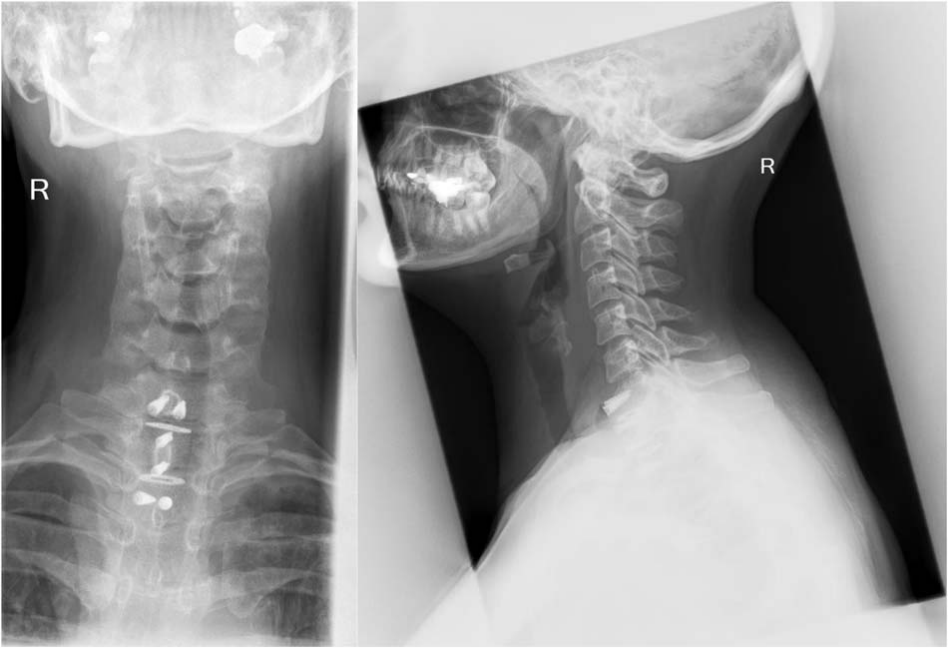
A 3-day postoperative X-ray of VBR and instrumentation in coronal (Right) and sagittal (Left) view.

**Figure 4:**
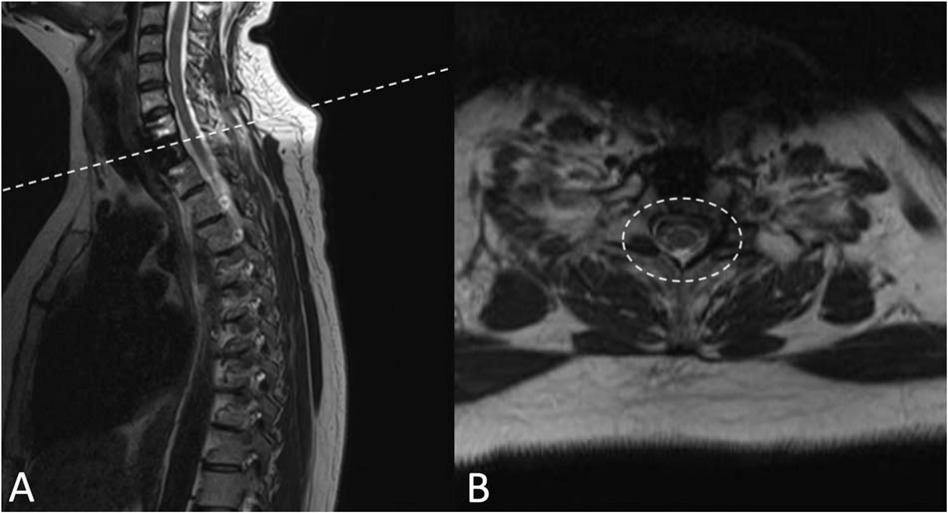
A 15-months postoperative follow up MRI Scan in May 2021. (**A**) Sagittal view of VBR cage indicating tumour free T2 body. (**B**) Transverse view of spinal compression reduction and resolved ESCC score.

**Figure 5:**
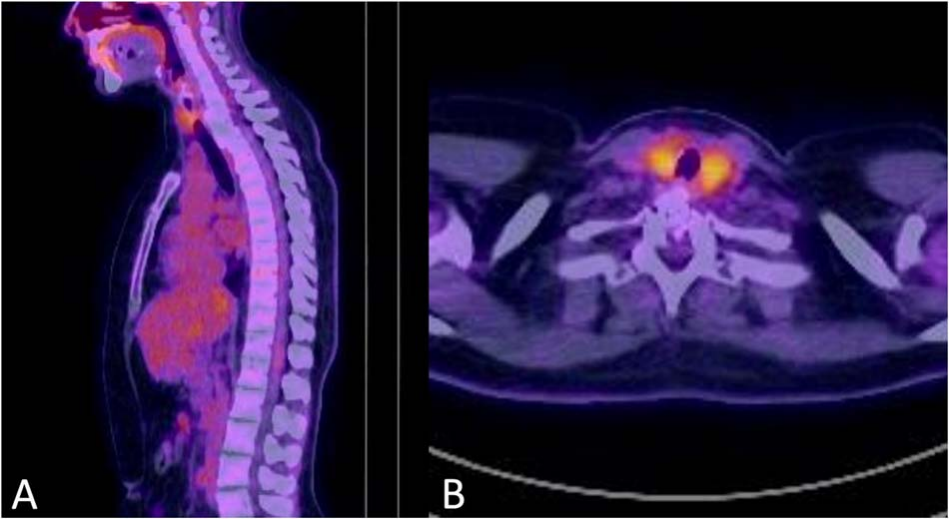
No signs of tumour recurrence at 30-months postoperative follow up PET-CT in July 2022.

There were no surgical complications intraoperatively or postoperatively. A 3-day postoperative X-ray determined there was no mispositioning of the VBR and instrumentation (Fig. 3). There were no inpatient medical complications and pain was managed appropriately. Patient was discharged from hospital shortly after the operation and recovery. As outpatient, she did not complain of pain or wasn’t immobilized outside of the hospital. A 15-month follow-up MRI and 30-month follow up Positron emission tomography–computed tomography (PET-CT) maintained to show that the patient was tumour free (Figs 4 and 5). Lastly, upon assessment in clinic over 30 months, the patient was fully mobile and self-reported to be pain free, living independently and comfortably. There were no hardware related issues and there wasn’t any development of infection.

## DISCUSSION

A high index of suspicion and understanding of the oligometastatic state allow for the patient to have radical oncological treatment. Having recognized that this is oligometastatic and metachronous. The chances of maximum local control with SBRT as primary treatment are recognized to be potentially curative. Barzilai et al. showed that local control rates of patients with OMD who were treated with SBRT alone were 92.3% at 3 months, 88.6% at 6 months and 84.8% at 1 year [12]. Another similar study Ho *et al*. looked at longer follow times showing that 1-, 2- and 5-year local progression rates were 85% (95% CI 68–94%), 82% (95% CI 64–91%) and 78% (95% CI 59–89%) [13]. However, this patient could not have radiosurgery as a primary treatment modality because the tumour was in contact with the spinal cord because of the risk of radiation myelitis. We described the different possible treatment options above. Conventional treatment of observation and waiting for the lady to develop neurological deficit before offering a palliative surgical procedure of decompression, stabilization and radiotherapy might maintain continence and mobility but will not allow radical control of tumour. She could have also undergone posterior decompression +/− stabilization but this also has palliative outcomes [14]. Posterior separation surgery can be considered and is the more common approach, however, in this patient there are several disadvantages of posterior separation surgery:

(1) Titanium plating and screws can cause artefact on scanning.
(2) Titanium cause reflection/deflection of radiotherapy beams.
(3) T1 in a female to have instrumentation requires Pedicle screws at C6, C7 that are difficult.
(4) Attempting corpectomy of T1 and inserting screws at T2/T3 from posterior is difficult.
(5) For a female, the scar is large and potentially leads to cervico–thoracic junction fusion.

To maximize local control and minimize morbidity, anterior separation corpectomy surgery was opted. The novel use of carbon fibre instrumentation offers high heat tolerance, high strength to weight ratio, resistance to corrosion & conductivity. Carbonaceous implants possess radiation properties like biological tissues and are more suitable for planning and delivering SBRT and disease surveillance. CT images are inherently more prone to artefacts which affects Hounsfield unit measurements. One of the most encountered CT artefacts, beam-hardening artefact, affects the measurement of radiodensity. Up until now it has been difficult to quantify how advantageous the radiolucency of CF pedicle screws is compared to titanium or metallic screws. In addition, there hasn’t been any signs of infection possibly due to the difficulty organisms have in order to produce a biofilm over carbon fibre as opposed to conventional titanium [15] The anterior approach is less invasive with less muscle dissection allowing earlier wound healing, earlier chemoradiotherapy and allowing easy access for SBRT. Anterior separation surgery at T1 should be considered over posterior separation surgery as it may offer a less invasive approach with less morbidity. Anterior separation surgery with postadjuvant SBRT is appropriate for metachronous breast oligometastatic disease. Carbon fibre VBR and instrumentation offers optimal SBRT planning and future surveillance, better infection control, high tensile strength, high heat resistance, resistance to corrosion and conductivity and is safe alternative to standard Titanium fixation. Anterior Spinal Separation Surgery with post adjuvant SBRT has shown maximum local control and can deliver curative, radical oncological control without the morbidity of Enneking radical surgery. The patient maintain disease free and pain free at 30 months postoperatively.

## Data Availability

The data underlying this article are available in the article and in its online supplementary material.
